# Correlation between retention and masticatory ability of magnetic attachment overdenture in elderly patients

**DOI:** 10.4314/ahs.v25i1.34

**Published:** 2025-03

**Authors:** Junmei Han

**Affiliations:** Department of Stomatology, Shijiazhuang fourth hospital, Shijiazhuang, Hebei, China

**Keywords:** Dentition defect, Denture restoration, Magnetic attachment retention repair, Denture retention and restoration with pole buckle attachment, Removable partial denture repair

## Abstract

**Background:**

To explore the correlation between restoration and retention of magnetic attachment overdenture and masticatory ability in elderly patients.

**Methods:**

200 elderly patients with most defects of dentition undergoing denture repair were selected, and with magnetic attachment retention repair in group A (n=70), with Taiji buckle attachment denture retention repair in group B (n=65), with removable partial denture repair in group C (n=65). The masticatory ability, abutment related indexes and the incidence of denture repair related complications in the three groups were compared, and the masticatory efficiency of patients with different magnetic attachment retention and repair effects before and after treatment was compared.

**Results:**

The bite force, retention and masticatory efficiency of group A were higher than group B and C (P<0.05). After treatment, gingival index, bleeding index and mobility in group A were higher than group B and C, plaque index in group B were higher than group A and C (P<0.05). The masticatory efficiency of patients with good retention and repair effect of magnetic attachment before and after treatment of patients with general effect were better than patients with poor effect and there was a positive correlation (r=0.320, 0.398, P<0.05).

**Conclusions:**

Magnetic attachment overdenture is effective and safe in most elderly patients with dentition defects.

## Introduction

Dentition defect is a common disease in stomatology, which refers to a kind of disease with incomplete permanent dentition caused by partial tooth loss. It is most common in the elderly group.

Dentition defect affects the normal chewing and auxiliary vocal function of elderly patients and seriously affects the quality of life of patients, so effective remedial measures should be taken actively. The clinical treatment methods of dentition defect are diversified^1^,^2^. At present, the more commonly used ones are removable partial denture repair, Taiji buckle attachment denture retention repair, magnetic attachment denture retention repair and so on, which have good therapeutic effects and can effectively improve the chewing ability and quality of life of patients. Different from traditional maxillofacial attachments, magnetic attachments can significantly improve the quality of denture repair and better meet the requirements of patients. As a long-term application prosthesis in the mouth, the retention and masticatory efficiency of denture repair has always been the focus of clinical attention and research. Dentition loss is a common problem in the elderly, which seriously affects the quality of life of patients. Based on this, this study investigated the correlation between retention and masticatory ability of magnetic attachment overdenture, in order to provide reference for clinical treatment of elderly patients with dentition defect.

## Materials and methods

### General information

200 elderly patients with most defects of dentition who underwent denture repair in Shijiazhuang fourth hospital from December 2018 to December 2019 were selected and divided into three groups according to different treatment methods. 70 patients with magnetic attachment retention repair were used as group A, 65 patients with Taiji buckle attachment denture retention repair were used as group B, and 65 patients with removable partial denture repair were used as group C. There was no significant difference in gender, age, weight, brushing times, combined diseases and types of dentition defects among the three groups (P>0.05, Table 1). This study was approved by the ethics committee of Shijiazhuang fourth hospital. Signed written informed consents were obtained from all participants before the study.

**Table 1:** Comparison of three groups of general data (min∼max, ±s)

Groups	A (n=70)	B (n=65)	C (n=65)	*t/χ* ^2^	*P*
Gender (Femal/Male, N)	32/38	35/30	33/32		
Age (Years)	59∼80(72.16±3.92)	58∼80(72.60±3.66)	58∼80(73.04±3.47)	0.957	0.386
Weight (Kg)	47∼82(64.19±8.59)	45∼85(66.27±9.36)	48∼83(65.45±8.71)	0.942	0.392
Brushing Frequency(/d)	0∼3(1.21±0.60)	0∼3(1.15±0.57)	0∼3(1.30±0.64)	1.017	0.364
Coronary Heart Disease	19(27.14)	15(23.08)	17(26.15)	0.326	0.854
Hypertension	26(37.14)	22(33.85)	25(38.46)	0.315	0.853
Diabetes	9(12.86)	12(18.46)	10(15.38)	0.809	0.667
Hyperlipidemia	14(20.00)	17(26.15)	13(20.00)	0.968	0.616
Class I	39(55.71)	35(53.85)	37(56.92)	0.127	0.939
Class II	31(44.29)	30(46.15)	28(43.08)		

### Inclusion criteria

I. All of them were Ken's class I and class II dentition defects. II. Age ≤ 80 years old. III. Good cognitive function without communication barrier. IV. No bad habits such as smoking and drinking. V. No osteoporosis. VI. Patients and their families are aware of this study and have signed the consent form.

### Exclusion criteria

I. Patients with hematological diseases. II. There is a history of infectious diseases near 3W3 weeks. III. Severe dysfunction of heart, brain, liver and kidney. IV. Physical weakness and serious reduction of daily living ability. V. Patients with autoimmune diseases and infectious diseases. VI. Patients with other oral diseases. VII. Patients with malignant tumors.

### Grouping method

Group A was treated with magnetic attachment retention restoration: 1∼3 teeth with symmetrical position were selected as attachment abutment teeth to ensure the balance of denture fixation. Sharp teeth with root length of 8mm were preferred as abutment teeth, and regular sequential periodontal treatment and root canal treatment were given to ensure that the alveolar bone absorption length did not exceed 1/2 of the root length. The operation specifications of magnetic attachment denture retention restoration were strictly followed, and they were tried on after abutment preparation and denture fabrication, The magnet is fixed in the base with self self-setting plastic and polished. Finish and polish.

Group B was treated with Taiji buckle attachment denture retention restoration: the abutment was treated with Taiji buckle attachment denture retention restoration. The abutment selection and tooth preparation process were the same as that of magnetic attachment denture retention restoration. First, the negative parts of the attachment were made, and then the impression and positive parts were made according to the position of the negative parts after trial wear and adjustment. Finally, the crown and attachment were bonded as a whole with adhesive, Avoid the penetration of adhesive into the negative and positive structures of the attachment.

Group C was repaired with removable partial denture: after replicating the model of oral and maxillofacial tissue morphology, titanium alloy was used to make stent removable denture, which was handed over to patients for trial wearing and further repaired. After prosthetic treatment, the three groups actively carried out health education to guide patients to do a good job in denture cleaning.

### Masticatory ability test method

Including occlusal force, retention force and masticatory efficiency. The MCF-8701 dental force tester produced by the medical school of Shanghai Jiaotong University was used to measure the maximum occlusal force of the patient. During the test, the occlusal piece was placed at the first molar, and the subject was asked to take the end sitting position and occlude as much as possible. The occlusal force was occluded 10 times continuously at a frequency of 2 Ss/time, and the average of the highest values of the three times was taken as the maximum occlusal force of the patient. The masticatory efficiency of the patients was measured by absorbance method. During the test, the patients chewed 2.0 g peanuts for 20 times on the left and right sides. The masticatory was collected and fixed to 1000 ml with double distilled water. After stirring for 1min and standing for 2 min, the middle and upper suspension was taken. The absorbance value was measured by spectrophotometer, and the average value of absorbance measured for 3 times was taken as the masticatory efficiency of the patients. In the study design, if attachment implant failure is found during follow-up, we will intervene in time and re-implant the patient free of charge.

### Observation index

I. masticatory ability before and after treatment, including bite force, retention and masticatory efficiency. II. The abutment related indexes of the three groups before and after treatment, including plaque index, gingival index, bleeding index and mobility, were measured by periodontal electronic pressure sensitive probe produced by Yeaple company in the United States. III. The incidence of denture repair related complications in group 3, including root caries, gingivitis, denture fracture, abutment fracture, magnet falling off and armature falling off. IV. Compare the masticatory efficiency of patients with different retention and repair effects of magnetic attachments before and after treatment, and explore the correlation between retention effect of magnetic attachments and masticatory ability. V. The retention effect of the three groups was evaluated from four aspects: aesthetics, fixation, masticatory ability and comfort. The total score was 0∼100. The patients were rated according to the specific situation. 85 and above were good, 70∼84 were average and below 70 were poor. VI. Stress distribution characteristics of magnetic attachment retention and repair. Make denture model sections, and cut 45 and 44 from the buccal lingual direction of the proximal and distal adjacent faces respectively. Each piece is 5.0∼5.5 mm thick, a total of 2 pieces. The test method is also based on the method of 8 pages in the literature. The observation points are marked as: a, b44 proximal and distal root tips, c, d45 proximal and distal root tips, e, f44 proximal and distal neck, g, h45 proximal and distal neck, and I, J, K and L are 1/4 of the missing tooth area from proximal to distal.

### Statistical analysis

The statistical analyses were performed using the Statistical Package for the Social Sciences version 22.0 (SPSS Inc., Chicago, IL, USA). Non-normally distributed metric variables were analyzed by the Kruskal–Wallis test and Mann–Whitney U-test. The Efficacy evaluation of the magnetic attachment overdenture between the same group (before and after the medical treatment) were analyzed by Wilcoxon's signed-rank test. P≤0.05 was considered statistically significant. Values were expressed as mean±standard deviation, unless stated otherwise.

## Results

Comparison of bite force and masticatory efficiency among the three groups There was no significant difference in bite force, retention and masticatory efficiency among the three groups before treatment (P>0.05). There were significant differences in bite force, retention and masticatory efficiency among the three groups after treatment (P<0.05), and group A>group B>group C (Table 2).

**Table 2:** Comparison of bite force, retention and masticatory efficiency among the three groups (±s)

Groups		A (n=70)	B (n=65)	C (n=65)	*t/χ* ^2^	*P*
Biting force	Pre-therapy	56.23±7.42	55.74±7.10	54.69±6.98	1.042	0.355
	Post-therapy	131.05±20.14	104.85±16.81	73.22±8.89	217.368	<0.001
Masticatory efficiency	Pre-therapy	0.25±0.05	0.27±0.07	0.26±0.08	1.483	0.22
	Post-therapy	0.89±0.08	0.67±0.08	0.38±0.05	856.711	9<0.001
Retentive force	Pre-therapy	0.43±0.05	0.45±0.07	0.43±0.06	2.413	0.092
	Post-therapy	2.93±0.25	2.40±0.22	1.98±0.19	310.571	<0.001

### Comparison of related indexes of abutment teeth in three groups

There was no significant difference in plaque index, gingival index, bleeding index and mobility between the three groups before treatment (P>0.05); There was significant difference in plaque index, gingival index, bleeding index and mobility among the three groups after treatment (P<0.05), and the three indexes of gingival index, bleeding index and mobility: group A>group B>group C, plaque index: group B>group A>group C (Table 3).

**Table 3:** Comparison of related indexes of three groups of abutments (±s)

Groups		A (n=70)	B (n=65)	C (n=65)	F	P
Plaque index	Pre-therapy	0.57±0.12	0.55±0.11	0.54±0.12	1.161	0.316
	Post-therapy	1.20+0.19	1.54±0.21	0.95±0.13	175.676	<0.001
Gingival index	Pre-therapy	0.19±0.07	0.18±0.06	0.20±0.05	1.758	0.175
	Post-therapy	0.71±0.08	0.59±0.07	0.35±0.04	516.022	<0.001
Bleeding index	Pre-therapy	0.31±0.06	0.30±0.05	0.32±0.06	2.005	0.138
	Post-therapy	0.74±0.08	0.61±0.07	0.41±0.05	398.967	<0.001
Looseness (mm)	Pre-therapy	0.39±0.07	0.42±0.08	0.40±0.07	2.896	0.058
	Post-therapy	0.98±0.10	0.82±0.05	0.53±0.06	635.08	<0.001

### Comparison of the incidence of denture repair related complications among the three groups

There was significant difference in the incidence of root caries, gingivitis, denture fracture, abutment fracture, magnet falling off and armature falling off among the three groups (P<0.05), and group A<group B<group C (Table 4).

**Table 4:** Comparison of related complications and retention effects of three groups of dentures (n, %)

Groups		A (n=70)	B (n=65)	C (n=65)		*P*
Complication	Root caries	3(4.29)	11(16.92)	23(35.38)	21.778	<0.001
	Denture fracture	2(2.86)	5(7.69)	15(23.08)	15.151	<0.001
	Marginal gingivitis	7(10.00)	14(21.54)	27(41.54)	18.699	<0.001
	Magnet falling off	2(2.86)	7(10.77)	24(36.92)	30.684	<0.001
	Abutment fracture	1(1.43)	6(9.23)	19(29.23)	24.244	<0.001
	Armature falling off	1(1.43)	5(7.69)	21(32.31)	30.302	<0.001
Retention effect	Good	50(71.43)	38(58.46)	25(38.46)	15.055	<0.001
	Commonly	18(25.71)	15(23.08)	20(30.77)	1.022	0.6
	Difference	2(2.86)	12(18.46)	20(70.77)	18.755	<0.001

### Retention effect

Compared with the retention effect of the three groups, the difference was statistically significant (P<0.05), and group a>group B>group C (Table 4).

### Correlation between retention and repair effect of magnetic attachment and masticatory ability

There was significant difference in masticatory efficiency between patients with different retention and repair effects of magnetic attachments before and after treatment (P<0.05), as shown in Table 5. The retention and repair effect of magnetic attachment was positively correlated with the masticatory efficiency before and after treatment (r=0.320, 0.398, P<0.05).

**Table 5:** Correlation between retention and repair effect of magnetic attachments and masticatory ability (±s)

Retention effect	Good	Commonly	Difference
n	50	18	2
Pre-therapy	0.26±0.04	0.24±0.03	0.18±0.02
Post-therapy	0.90±0.04^#^	0.87±0.05^#^	0.82±0.06^#^
*P*	<0.005	<0.005	<0.005

Compared with before treatment: P<0.05

### Stress distribution characteristics of magnetic attachment retention and repair

**Figure 1:**
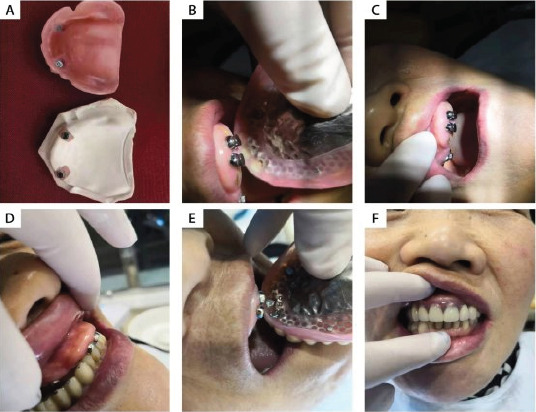
Follow up results after magnetic attachment overdenture

The vertical force was greater than the lateral force in different sections of elderly patients with magnetic attachment retention and repair, and the difference was statistically significant (P<0.05, Table 6 and Figure 1).

**Table 6:** Characteristics of stress distribution in retention and repair of magnetic attachments (±s, N/mm)

Section site	Vertical force	Lateral force	t	P
a	50.41±5.27	30.69±3.98	24.983	<0.001
b	54.32±6.12	32.84±4.03	24.525	<0.001
c	57.28±6.34	33.56±4.26	25.982	<0.001
d	59.31±5.74	35.67±4.4 1	27.324	<0.001
e	57.25±5.82	35.41±4.58	24.673	<0.001
f	59.63±6.02	37.87±4.96	23.340	<0.001
g	65.37±7.12	38.96±4.82	25.699	<0.001
h	70.12±7.69	40.12±5.19	27.055	<0.001
i	59.62±5.14	38.94±5.69	27.270	<0.001
j	59.62±5.84	36.12±5.12	25.316	<0.001
k	54.31±6.12	33.96±4.74	22.636	<0.001
l	51.37±5.63	31.84±4.35	22.966	<0.001

## Discussion

The main causes of dentition defect are caries, periodontal disease, trauma, jaw defect, developmental disorder, etc. The incidence of dentition defect is high in elderly patients due to risk factors such as age, periodontal disease, poor oral hygiene and poor eating habits^3^,^4^. Prosthetic treatment includes fixed denture, removable partial denture and implant denture. The retention and stability of overdenture has always been a clinical concern^5^.

Removable partial denture restoration uses natural teeth, mucosa and bone support to fix the denture in the dentition through retainer snap ring and base. Patients can take off and wear and clean the denture according to their needs. It has wide adaptability, high safety and convenient use, but it has the disadvantage of poor retention^6^,^7^. Taiji clasp attachment denture retention repair and magnetic attachment denture retention repair are new technologies developed in recent years to repair the loss of dentition. The processing technology and operation process of the denture are relatively simple, and they are of positive significance to protect the abutment and periodontal tissue and maintain the stability of the denture. Taiji clasp attachment denture is a kind of elastic denture attachment denture. The special latch design can reduce the stress of abutment teeth, which is of great significance for the protection of abutment teeth. It is suitable for the treatment of Ken jade and domain dentition defects^8^-^10^. Previous studies have confirmed that Taiji clasp attachment denture retention repair can achieve good results in the treatment of patients with dentition defects. However, Taiji clasp can not be used in all unilateral free deletions. The selection of intermaxillary distance, the conditions of abutment teeth, the thickness of distal alveolar bone and mucosal elasticity need to be carefully considered, and its clinical application has certain limitations. At present, magnetic attachment has been gradually applied to various oral and maxillofacial restorations^11^-^13^. Magnetic attachment is a device that uses magnetic force to connect the denture to the abutment in order to achieve retention and stability. It has the characteristics of simple operation, self-regulation, reduction, relatively free lateral movement and less trauma to the residual root. It solves the problems of poor retention of the traditional complete overdenture Low masticatory efficiency and unsightly clasp retainer of removable partial denture. Traditional magnetic attachments have disadvantages such as poor corrosion resistance, which limit their clinical application^14^,^15^.

With the development of new magnetic materials, magnetic attachments use the adsorption between magnets at denture and abutment to fix denture on implant or abutment, which has become a new retention technology in the field of prosthodontics^16^,^17^. As a long-term prosthetic used in oral cavity, good retention ability and masticatory efficiency are important indicators to measure its superiority over other prosthetic methods. This study found that there was a positive correlation between the retention and repair effect of magnetic attachment and masticatory efficiency before and after treatment^18^,^19^. Scanning masticatory efficiency is the key index to evaluate the retention and repair effect of magnetic attachment. The magnetic attachment overdenture can achieve better bite force, retention and masticatory efficiency than removable partial denture and Taiji buckle attachment denture. The reason for the analysis is that the magnetic attachment obtains the retention force through the magnetic attraction of the magnet structure placed in the tissue plane of the denture and the connecting structure placed on the abutment^20^-^23^. Because the magnetic attachment has the advantages of unrestricted direction, it is applicable to any part in principle. On the basis of inheriting the original advantages and functions of the overdenture, the magnetic attachment overdenture gives a certain retention force between the abutment and the denture. It retains the physiological stimulation to alveolar bone and slows down the absorption rate of alveolar ridge, so as to significantly enhance the retention and stability of denture, improve the bite force and chewing efficiency of patients, and improve the rate of good retention effect. Ma Tianchi and other researchers pointed out that the magnetic attachment denture largely retains the residual roots and crowns that may be removed by other repair methods, and can provide good denture retention and support, obtain better chewing effect, avoid severe alveolar bone absorption, and improve the chewing function of patients^24^,^25^.

Further confirm the correctness of the results of this study. And in the results of this study, the effect of magnetic attachment overdenture repair in protecting gingiva, preventing bleeding and stabilizing denture is better than that of removable partial denture repair and Taiji buckle attachment denture retention repair. The clinical effect is better, but the plaque index is at a high level after treatment, suggesting that denture cleaning should be strengthened in clinical work^26^-^28^. In this study, by guiding the patients to clean the denture every day, it was found that the incidence of root caries, gingivitis, denture fracture, abutment fracture, magnet falling off and armature falling off in the treatment of magnetic attachment covered denture were low and had high safety.

Relevant reports show that the retention force of magnetic attachment overdenture is slightly worse than that of rod clamp attachment, but its stress is relatively uniform, and satisfactory retention and stability can still be obtained.

This study found that the vertical force of different sections in elderly patients with magnetic attachment retention restoration is greater than the lateral force, suggesting that the magnetic attachment denture can move by itself when subjected to a large lateral force, and the torsional force on the abutment is small, which can ensure the uniform distribution of stress around the abutment, avoid greater compression on the alveolar bone, delay the absorption of alveolar ridge and prevent the atrophy of periodontal tissue. The limitation of this study is that the sample size of the study is small, the evidence results do not meet the ideal state of the theory, and the sample size needs to be further expanded for research.

## Conclusion

The use of magnetic attachment overdenture in the treatment of most elderly patients with dentition defects has a positive correlation between repair retention and masticatory ability, which can significantly improve the bite force and masticatory efficiency of patients, reduce the stress of bone tissue around abutment teeth, protect gums, prevent bleeding, stabilize dentures and reduce the incidence of complications.
